# Predictive value of systemic immune-inflammatory biomarkers for drug-induced liver injury in hepatitis B virus surface antigen positive tuberculosis patients: A retrospective observational study

**DOI:** 10.1097/MD.0000000000040349

**Published:** 2024-11-08

**Authors:** Haiping Dong, Jingyuan Feng, Xinwei Chang, Shaoling Wu, Guidan Tang, Feng Liang, Haojie Tang, Yaping Dong, Weiming Fang, Jinxing Hu, Weiyong Wang

**Affiliations:** a Guangzhou Key Laboratory of Tuberculosis Research, Department of Tuberculosis, Guangzhou Chest Hospital, State Key Laboratory of Respiratory Disease, Guangzhou, China; b Department of Clinical Medicine, Guangzhou Medical University, Guangzhou, China; c Breast Tumor Center, Sun Yat-Sen Memorial Hospital, Sun Yat-Sen University, Guangzhou, China.

**Keywords:** biomarkers, CD4/CD8, EOS%, HBsAg, liver injury, MLR, NLR, SII

## Abstract

Drug-induced liver injury (DILI) is a major concern in tuberculosis (TB) treatment. For early detection of DILI, immune-inflammatory biomarkers are needed for better management. To explore the predictive effect of systemic immune-inflammation index (SII) combined with neutrophil-to-lymphocyte ratio (NLR), platelet-to-lymphocyte ratio (PLR), monocyte-to-lymphocyte ratio (MLR), eosinophil (EOS%), and CD4/CD8 on DILI occurrence in TB patients with HBsAg positive. This is a retrospective study enrolling patients who were treated with anti-tuberculosis drugs and infected with hepatitis B virus (HBV) in the Guangzhou Chest Hospital from 2018 to 2023. Population demographics and clinical data of 2643 patients were collected by reviewing electronic medical records. Using a propensity score matching model, the study ultimately included 516 patients (258 patients with DILI and 258 patients without DILI). Logistic regression analysis was conducted to investigate the predictive role of systemic immune-inflammatory biomarkers (SII, NLR, MLR, EOS%, and CD4/CD8) in DILI in hepatitis B virus surface antigen-positive TB patients (HBV-TB-DILI). As compared to patients without DILI, patients with DILI have elevated levels of systemic immune-inflammatory biomarkers (SII, NLR, MLR, EOS%, and CD4/CD8), (all *P* < .05). The SII, NLR, MLR, PLR, EOS%, and CD4/CD8 are risk factors of HBV-TB-DILI. The NLR, MLR, SII, and EOS% were positively correlated with liver function (*P* < .001). The combination of SII, NLR, MLR, EOS%, and CD4/CD8 demonstrated good predictive performance for DILI occurrence in HBV-TB patients. The combination of SII, NLR, MLR, EOS%, and CD4/CD8 demonstrated good predictive performance for DILI occurrence in HBV-TB patients.

## 1. Introduction

Tuberculosis (TB) is caused by the mycobacterium tuberculosis complex (MTBC) and is one of the oldest diseases as well as a major cause of death worldwide.^[1]^ Globally, the TB incidence rate reversed the approximately 2% annual decline observed over the past 20 years.^[2]^ The World Health Organization (WHO) recommends a standard anti-tuberculosis treatment regimen that includes Isoniazid (INH), Rifampin (RIF), and Pyrazinamide (PZA), which are associated with high liver toxicity. Anti-tuberculosis drug-induced liver injury (AT-DILI) is a common side effect, potentially leading to acute liver failure (ALF), even death, and requiring liver transplantation.^[3]^ Severe AT-DILI can also result in drug resistance and treatment interruption. In China, anti-tuberculosis drugs are the second leading cause of drug-induced liver injury (DILI). In tuberculosis-endemic regions of India, AT-DILI is associated with 5% to 25.7% of ALF patients.^[4]^

Chronic hepatitis B (CHB) is the most prevalent form of chronic viral hepatitis.^[5]^ Approximately 240 million people worldwide are chronically infected with hepatitis B virus (HBV). The treatment of co-infection with tuberculosis and CHB presents significant challenges.^[6]^ TB patients often share similar epidemiological characteristics and risk factors with HBV infection.^[7]^ Despite biological differences, HBV and TB exhibit a unique symbiotic relationship.^[8]^ Recent meta-analyses show that the overall prevalence of HBV among adults with TB is 7.1%.^[9]^ HBV co-infection increases the risk of AT-DILI and is associated with poor treatment outcomes.^[6]^ The hepatotoxicity of anti-tuberculosis drugs and immune liver injury caused by HBV replication exacerbate liver dysfunction. High HBV DNA load is also a critical risk factor for liver failure.

The systemic immune-inflammation index (SII) is a newly developed comprehensive inflammatory biomarker that was introduced by Hu et al in 2014. It provides insights into both the localized immune responses and the systemic inflammation occurring in the entire body. Previous studies have utilized SII to forecast and evaluate the prognosis of different types of solid tumors, including gastric cancer, non-small cell lung cancer, pancreatic cancer, and esophageal cancer. Furthermore, SII has demonstrated a strong predictive capability for determining the prognosis of cardiovascular diseases. The SII is a composite index that takes into account platelet count (PLT), absolute neutrophil count (NEU), and absolute lymphocyte count (LYM), thereby offering a more comprehensive assessment of the body’s inflammatory and immune status. This index serves as a novel biological marker for predicting clinical outcomes.

Liver inflammation is a prevalent occurrence in cases of DILI. Drugs have the ability to create adducts with endogenous proteins, which then act as novel antigens. When these drug-protein adducts (new antigens) are internalized by antigen-presenting cells (APCs) and subsequently presented to T cells on major histocompatibility complex (MHC) class II proteins, they have the potential to initiate subsequent adaptive immune responses.

There is growing evidence supporting the inflammation stress hypothesis of liver damage. This hypothesis suggests that the inflammation that may occur during drug therapy can interact with the effects of the compound and result in liver damage. However, the relationship between SII and DILI in patients with HBV and TB is still not well understood. Our study aims to investigate the relationship between systemic immune-inflammatory biomarkers and DILI in hepatitis B virus surface antigen-positive TB patients (HBV-TB-DILI). The early detection of DILI in TB patients who are positive for HBsAg can assist clinicians in adjusting HBV and TB treatment plans (such as initiating antiviral therapy earlier or switching to alternative TB treatment options) in order to minimize the occurrence of DILI and prevent negative patient outcomes.^[10]^

## 2. Materials and methods

### 2.1. Study design and setting

We conducted a retrospective study from 2018 to 2023 to investigate the demographic, clinical, and laboratory features and outcomes of inpatients with AT-DILI who had received multidrug therapy for active TB at the Guangzhou Chest Hospital. A total of 3376 inpatients were treated with anti-TB medication. The study complied with the relevant principles of the Declaration of Helsinki, and was approved by the Guangzhou Chest Hospital (KY-2024-007). Written informed consent was obtained from each participant or his/her legal representatives.

### 2.2. Study populations

We retrospectively enrolled patients diagnosed with TB who received anti-tuberculous agents at the Guangzhou Chest Hospital from January 1, 2018, to December 31, 2023.

All patients enrolled were diagnosed for HBsAg positive. Liver ultrasonography and fibroscans were carried out to exclude patients with liver cirrhosis. Pregnant patients or patients with abnormal baseline liver function tests (LFT) due to congestive heart failure, diabetes mellitus, severe chronic kidney disease, autoimmune disease, hepatic malignancy or alcohol abuse were excluded from this study. Serological screening of antibodies against hepatitis C virus (HCV), hepatitis A virus (HAV), hepatitis E virus (HEV) and human immunodeficiency virus (HIV) was carried out for all patients to eliminate positive cases. This article was compiled after obtaining the written informed consent of the closest relatives of the patient.

### 2.3. Definition

Laboratory results of all TB cases were screened retrospectively to identify patients with a rise in ALT of greater than 3 multiples of the upper limit of normal (ULN) or a rise in total bilirubin of greater than 2 multiples of the ULN. The clinical records of the positive screens were then scrutinized to establish a diagnosis of TB-DILI. The TB-DILI, in accordance with American Thoracic Society (ATS) criteria, was defined by an alanine transaminase (ALT) greater than 3 multiples of the ULN in the presence of typical hepatitis symptoms or an ALT greater than 5 multiples of the ULN or a rise in total bilirubin greater than 2 times the ULN regardless of symptoms in the absence of an alternative explanation. Only cases considered at the time to be TB-DILI by the treating clinician were included. The Roussel Uclaf causality assessment method (RUCAM) was performed as an objective assessment of likelihood that the anti-tuberculosis treatment (ATT) was responsible for the liver test (LT) abnormalities.

HBV infection was defined as a positive HBsAg.

Severity of AT-DILI is defined as follows: besides elevated ALT or alkaline phosphatase (ALP) levels reaching criteria for DILI (ALT ≥ 3 ULN, ALP ≥ 2 ULN), severities of DILI were defined as: Grade-1 if total bilirubin (TBIL) was < 2.5ULN and international normalized ratio (INR) < 1.5; Grade-2 if TBIL was ≥ 2.5 ULN and INR ≥ 1.5; Grade-3 if TBIL was ≥ 5 ULN with or without INR ≥ 1.5; Grade-4 if TBIL ≥ 10 ULN, INR ≥ 2.0 or partial thromboplastin activity (PTA) < 40%, possibly accompanied by ascites, hepatic encephalopathy, or other organ failures related to DILI.

These counts were used to determine the inflammatory biomarkers listed below: systemic immune-inflammation index (SII = PLT × [NEU/LYM]), neutrophil-to-lymphocyte ratio (NLR = NEU/LYM), monocytes-to-lymphocyte ratio (MLR = MONO/LYM).

### 2.4. Outcomes

The primary outcome is the occurrence of DILI in HBV-TB patients. The main objective of this study is to explore the correlation between inflammatory biomarkers based on peripheral complete blood count (CBC) counts and the occurrence of DILI in HBV-TB patients, as well as to understand the predictive value of inflammation-related markers for DILI in HBV-TB patients.

### 2.5. Study variables

The baseline for data collection was defined as the end of the second month after initiating anti-TB treatment. The date used was either the date of TB diagnosis or the start date of anti-TB treatment, whichever was earlier. Demographic, clinical, and laboratory data were collected at baseline.

### 2.6. Blood analysis

The study analyzed whole blood cell counts and classifications from peripheral blood samples. Blood samples were collected in calcium-EDTA anticoagulant tubes.

### 2.7. Statistical analysis

The study population was determined by adjusting for propensity scores, which included baseline demographic, clinical, and laboratory variables. Continuous variables were presented as the mean ± SD and compared between the groups using “*t*” test. A chi-square test was applied for categorical variables and was presented as percentages. The odds ratio (OR), 95% confidence interval (CI), and multiple logistic regression were used to assess the risk factors associated with DILI in HBV-TB patients. Linear correlation analysis was used to explore the correlation between inflammatory markers and liver function indicators.

Youden index refers to sensitivity + specificity − 1 in receiver operating characteristic (ROC) curve. The optimal cutoff for predicting DILI was defined by creating a ROC curve to yield the highest Youden index value. Potential factors associated with different levels of hepatotoxicity in the mild liver injury group, moderate liver injury group, severe liver injury group, and liver failure group were analyzed by 1-way analysis of variance (ANOVA). Data were processed using SPSS 25.0 (IBM SPSS Statistics for Windows, version 25.0, Armonk). All statistical tests were 2-tailed. *P* values <.05 were considered significant.

## 3. Results

### 3.1. Comparison of patients with and without DILI

From 2018 to 2023, a total of 2643 cases met the inclusion criteria. Among them, 339 developed DILI, while 2304 did not. A propensity score matching model was applied to match the 2 groups. Ultimately, this study comprised a total of 516 patients, with 258 individuals assigned to the liver damage group and 258 individuals assigned to the non-liver damage group. A comparison of the baseline characteristics between 2 groups is presented in Table 1.

**Table 1:** Comparison of general data between patients with and without DILI.

Index	Patients with DILI (n = 258)	Patients without DILI (n = 258)	*P* value
Gender			.032*
Male	70	210	
Female	188	48	
Age (yr)	50.67 ± 14.42	53.07 ± 13.43	.051
WBC	7.61 ± 3.63	6.77 ± 2.96	.004*
HGB	116.36 ± 23.71	124.34 ± 24.29	＜.001*
EOS%	4.39 ± 4.80	2.10 ± 3.12	＜.001*
ALB	31.96 ± 6.52	35.99 ± 6.63	＜.001*
ALT	111.40 ± 206.14	47.02 ± 190.30	＜.001*
AST	103.07 ± 200.61	53.41 ± 243.48	.012*
ALP	129.63 ± 111.77	105.19 ± 78.55	.089*
GGT	114.32 ± 119.88	75.08 ± 126.76	＜.001*
ADA	23.98 ± 13.47	16.70 ± 8.67	＜.001*
CHE	4844.73 ± 2521.44	5827.24 ± 2716.70	.012*
DBIL	12.39 ± 19.58	8.27 ± 26.58	.045*
TBIL	19.69 ± 23.42	13.57 ± 33.09	.016*
TBA	36.59 ± 43.11	20.39 ± 43.95	.013*
FIB	3.08 ± 1.37	3.70 ± 1.47	＜.001*
*APTT	29.52 ± 5.43	29.43 ± 11.14	.916
CRP	4.39 ± 4.79	2.11 ± 3.12	＜.001*
PCT	4.39 ± 4.79	2.11 ± 3.12	＜.001*
CD4/CD8	1.99 ± 1.10	1.55 ± 0.69	＜.001*
NLR	6.62 ± 10.95	3.94 ± 11.95	＜.001*
MLR	0.74 ± 2.03	0.40 ± 0.36	.009*
PLR	284.62 ± 456.28	165.85 ± 110.77	＜.001*
SII	1730.59 ± 2350.55	796.08 ± 857.14	＜.001*
SIRI	4.42 ± 7.87	2.11 ± 3.12	＜.001*
Cr	89.61 ± 260.96	74.01 ± 46.76	.344
BUN	5.13 ± 2.33	5.00 ± 3.92	.663
Complications			.878
With	76 (29.57%)	75 (28.96%)	
Without	181 (70.43%	184 (71.04%)	

Complications: hypoalbuminemia, ascites, jaundice, portal hypertension, esophageal and gastric varices, hepatic encephalopathy and cirrhosis.

ADA = adenosine deaminase, ALB = albumin, ALP = alkaline phosphatase, ALT = alanine aminotransferase, APTT = activated partial thromboplastin time, AST = aspartate aminotransferase, BUN = blood urea nitrogen, CD4/CD8 = CD4 to CD8 ratio, CHE = cholinesterase, Cr = creatinine, CRP = C-reactive protein, DBIL = direct bilirubin, EOS% = eosinophil percentage, FIB = fibrinogen, GGT = gamma-glutamyl transpeptidase, HGB = hemoglobin, MLR = monocyte to lymphocyte ratio, NLR = neutrophil to lymphocyte ratio, PCT = procalcitonin, PLR = platelet to lymphocyte ratio, SII = systemic immune-inflammation index, SIRI = simplified immune-inflammation index, TBA = total bile acids, TBIL = total bilirubin, WBC = white blood cell count,

* Statistically significant (*P* < .05).

The groups with and without DILI comprised 188 and 210 males, and 70 and 48 females, respectively, with statistically significant differences (*P* < .05). There were no significant differences in terms of patient age and comorbidity rates between the group with liver damage and the group without liver damage. Compared to the group without liver damage, the group with liver damage demonstrated lower levels of hemoglobin (HB), albumin (ALB), cholinesterase (CHE) and fibrinogen (FIB). There were no significant differences between the liver damage group and the non-liver damage group in terms of creatinine (Cr), blood urea nitrogen (BUN) and activated partial thromboplastin time (APTT). In comparison to the group without liver damage, the group with liver damage exhibited higher levels of white blood cell count (WBC), eosinophil percentage (EOS%), C-reactive protein (CRP)，procalcitonin (PCT)，CD4/CD8, neutrophil to lymphocyte ratio (NLR), monocyte to lymphocyte ratio (MLR), platelet to lymphocyte ratio (PLR), systemic immune-inflammation index (SII) and simplified immune-inflammation index (SIRI).

There was no statistically significant difference between the 2 groups in terms of alkaline phosphatase (ALP). However, the differences in alanine aminotransferase (ALT), aspartate aminotransferase (AST), gamma-glutamyl transpeptidase (GGT), adenosine deaminase (ADA) were all found to be statistically significant (all *P* < .05). Age, Cr, BUN, APTT, ALP, and comorbidity rates did not show any statistically significant differences between the liver damage group and the non-liver damage group (refer to Table 1). Compared to the group without liver damage, the group with liver damage demonstrated increased levels of WBC, EOS%, CRP, PCT, CD4/CD8, NLR, MLR, PLR, SII, and SIRI, while exhibiting decreased levels of HB, ALB, CHE and FIB. The complications observed in this group included hypoalbuminemia, ascites, jaundice, portal hypertension, esophageal and gastric varices, hepatic encephalopathy and cirrhosis (Table 1).

### 3.2. Correlation between inflammatory markers and liver function indicators

**Figure 1.**
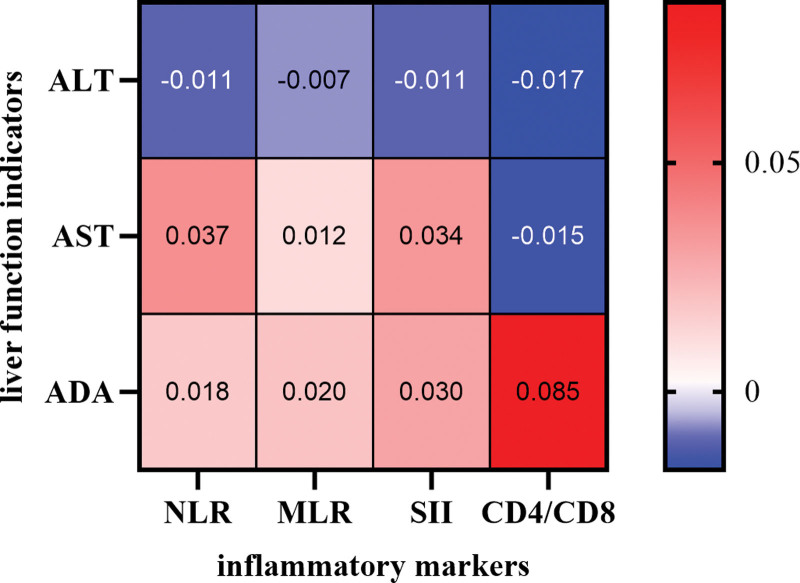
Pearson correlation analysis between inflammatory markers and liver function indicators, *P* value < .001 indicating a highly significant correlation. ADA = adenosine deaminase, ALT = alanine aminotransferase, AST = aspartate aminotransferase, MLR = monocyte to lymphocyte ratio, NLR = neutrophil to lymphocyte ratio, SII = systemic immune-inflammation index.

Pearson correlation indicates significant positive correlations between NLR, MLR, SII, CD4/CD8, and liver function (Fig. 1).

### 3.3. Association of SII, NLR, MLR, EOS% and CD4/CD8 with HBV-TB-DILI risk

We performed a logistic regression analysis, which included inflammation-related indicators, to identify independent predictors of DILI in patients with HBV and TB. The results of this analysis are presented in Table 2. According to the univariate binary logistic regression analysis, NLR, MLR, EOS%, CD4/CD8, and SII were found to be independent predictors of HBV-TB-DILI. Similarly, the multivariable binary logistic regression analysis confirmed that NLR, MLR, EOS%, CD4/CD8 ratio, and SII were all independent predictors of HBV-TB-DILI (Table 2).

**Table 2:** Predictors of the occurrence of DILI in HBV-TB patients by logistic regression analyses.

Variable	Univariate		Multivariate	
	OR (95% CI)	*P* value	OR (95% CI)	*P* value
Gender	1.573 (1.038–2.384)	.033*		
WBC	1.083 (1.024–1.146)	.005*		
EOS%	1.267 (1.171–1.372)	＜.001*	1.25 (1.138–1.373)	＜.001*
ALT	1.006 (1.003–1.009)	＜.001*		
AST	1.003 (1.001–1.006)	.005*		
GGT	1.003 (1.001–1.005)	.001*		
ADA	1.078 (1.054–1.102)	＜.001*		
ALB	0.91 (0.884–0.937)	＜.001*		
CRP	1.266 (1.17–1.37)	＜.001*		
PCT	1.266 (1.17–1.37)	＜.001*		
CD4/CD8	1.866 (1.422–2.448)	＜.001*	1.606 (1.193–2.161)	.002
NLR	1.092 (1.045–1.141)	＜.001*	0.883 (0.804–0.968)	.008
MLR	6.882 (3.639–13.016)	＜.001*	5.341 (1.572–18.15)	.007
PLR	1.005 (1.003–1.006)	＜.001*		
SII	1.001 (1–1.001)	＜.001*	1.001 (1–1.001)	＜.001*
SIRI	1.167 (1.093–1.246)	＜.001*	0.915 (0.798–1.049)	.204

ADA = adenosine deaminase, ALB = albumin, ALT = alanine aminotransferase, AST = aspartate aminotransferase, CD4/CD8 = CD4 to CD8 ratio, CRP = C-reactive protein, EOS% = eosinophil percentage, GGT = gamma-glutamyl transpeptidase, MLR = monocyte to lymphocyte ratio, NLR = neutrophil to lymphocyte ratio, PCT = procalcitonin, PLR = platelet to lymphocyte ratio, SII = systemic immune-inflammation index, SIRI = simplified immune-inflammation index, WBC = white blood cell count.

* Statistically significant (*P* < .05).

### 3.4. ROC curve analysis for DILI in HBV-TB patients

**Figure 2.**
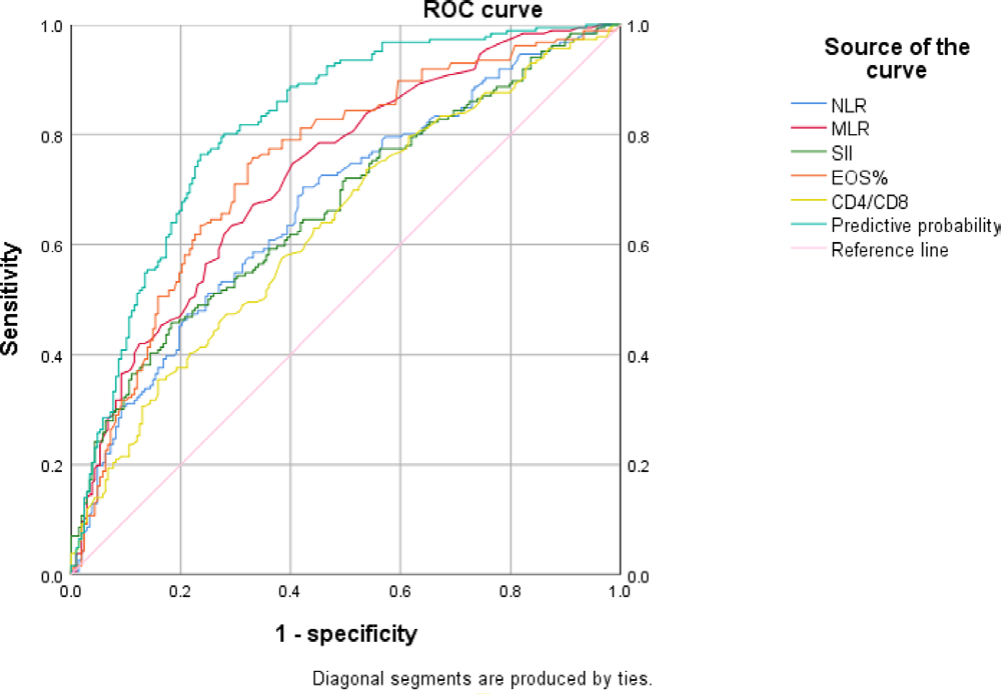
Predictive value of NLR、MLR、SII、EOS% and CD4/CD8 for prognosis of TB-DILI patients. EOS% = eosinophil percentage, MLR = monocyte to lymphocyte ratio, NLR = neutrophil to lymphocyte ratio, SII = systemic immune-inflammation index, TB-DILI = tuberculosis drug-induced liver injury.

ROC curve analyses for NLR, MLR, EOS%, CD4/CD8 and SII to predict DILI occurrence in HBV-TB patients demonstrated an AUC value of 0.671 (95% CI: 0.617–0.724，*P* < .001) with a positive likelihood ratio of 1.649 for the NLR、AUC value of 0.729 (95% CI: 0.680–0.778，*P* < .001) with a positive likelihood ratio of 1.868 for MLR、AUC value of 0.744 (95% CI: 0.695–0.793，*P* < .001)with a positive likelihood ratio of 0.264 for EOS% and AUC value of CD4/CD8 is 0.635 (95% CI: 0.580–0.689, *P* < .001) with a positive likelihood ratio of 1.377 for CD3/CD4. The AUC for SII was 0.668 (95% CI: 0.614–0.721，*P* < .001)with a positive likelihood ratio of 2.473. The cutoff value of the SII (1166.505) was associated with 45.5% sensitivity and 81.6% specificity (Fig. 2). In addition, the cutoff value of NLR (2.925) was associated with 70.1% sensitivity and 57.5% specificity, the cutoff value of MLR (0.355) was associated with 74.9% sensitivity and 59.9% specificity, the cutoff value of EOS% (1.78) was associated with 75.4% sensitivity and 66.7% specificity, and the cutoff value of CD4/CD8 (1.365) was associated with 73.8% sensitivity and 46.4% specificity (Fig. 2).

### 3.5. Risk factors associated with DILI grading

The statistical analysis between grades of DILI showed significant differences in ALT (*F* = 4.93) and AST (*F* = 6.079; *P* < .05). However, NLR, MLR, SII, EOS%, CD4/CD8, ALP, and ADA did not show statistical significance (Table 3).

**Table 3:** Risk factors associated with drug-induced liver injury grading.

Variable	Liver function grading	N	M ± SD	*F*	*P*
NLR	1	228	6.66 ± 11.50	0.22	.88
	2	21	6.53 ± 4.91		
	3	4	1.82 ± 0.54		
	4	5	8.13 ± 5.64		
MLR	1	228	0.75 ± 2.16	0.08	.97
	2	21	0.64 ± 0.29		
	3	4	0.28 ± 0.10		
	4	5	0.93 ± 0.58		
SII	1	228	1761.08 ± 1527.56	0.446	.721
	2	21	1577.87 ± 1390.59		
	3	4	245.34 ± 128.29		
	4	5	1885.36 ± 1558.96		
EOS%	1	228	4.19 ± 4.37	1.91	.13
	2	21	6.75 ± 3.31		
	3	4	4.57 ± 2.95		
	4	5	3.44 ± 2.53		
CD4/CD8	1	228	2.02 ± 1.85	0.968	.409
	2	21	1.77 ± 1.72		
	3	4	0.48 ± 1.55		
	4	5	2.11 ± 1.29		
ALT	1	228	96.84 ± 118.73	4.93	.002 *
	2	21	251.26 ± 581.94		
	3	4	319.60 ± 386.86		
	4	5	63.10 ± 37.06		
AST	1	228	85.68 ± 107.00	6.079	.001 *
	2	21	266.68 ± 586.44		
	3	4	243.70 ± 234.27		
	4	5	124.44 ± 75.24		
ALP	1	228	122.83 ± 112.39	0.786	.459
	2	21	161.29 ± 117.38		
	3	4	121 ± 98.36		
	4	5	158.4 ± 87.54		
ADA	1	228	23.49 ± 13.77	1	.393
	2	21	27.24 ± 11.07		
	3	4	27.03 ± 6.53		
	4	5	30.88 ± 9.03		

ADA = adenosine deaminase, ALP = alkaline phosphatase, ALT = alanine aminotransferase, AST = aspartate aminotransferase, CD4/CD8 = CD4 to CD8 ratio, CI = confidence interval, EOS% = eosinophil percentage, *F* = ratio of variances, MLR = monocyte to lymphocyte ratio, NLR = neutrophil to lymphocyte ratio, SII = systemic immune-inflammation index.

## 4. Discussion

This study indicates that the SII, a novel biomarker incorporating NEU, PLT, and LYM counts, is an independent predictor of DILI in HBV-TB patients. Furthermore, SII demonstrates superior predictive capability over the NLR for DILI in HBV-TB patients. This is the first literature report evaluating the predictive power of SII and comparing it with NLR and MLR in the context of DILI risk in HBV-TB patients.

The SII is a composite index based on PLT, NEU, and LYM counts, providing a more comprehensive reflection of inflammation and immune status.^[11]^ It is a novel biomarker used for predicting clinical outcomes. Another study supports this finding, suggesting that SII is a powerful tool for predicting the progression of various diseases.^[12]^ The NLR, as a simple biomarker obtainable through routine clinical tests,^[13]^ has been reported to reflect systemic inflammation and play a positive role in assessing patient condition and predicting prognosis.^[14]^ Both NEU and LYM are involved in immune defense mechanisms, and NLR is more stable in disease evaluation compared to single parameters.^[15]^ The MLR, based on peripheral MONO and LYM, is another effective new prognostic factor that can predict the prognosis and treatment response of certain malignancies.^[16]^

In our study, we evaluated for the first time the relationship between SII, NLR, and MLR with HBV-TB-related DILI. The results indicate that SII, NLR, and MLR are significantly elevated in HBV-TB patients following the onset of DILI. The degree of inflammation at the onset of infection is clinically reflected by CRP, WBC, and PCT indices.^[17]^ Concurrently, our study found elevated levels of CRP, WBC, and PCT in patients in the case group of this cohort.

A novel in vitro diagnostic method for DILI has recently been proposed, which utilizes monocyte-derived hepatocyte-like (MH) cells to identify drugs that cause DILI. The principle of this method is based on the release of damage-associated molecular patterns (DAMPs) from damaged liver cells, which activate the innate immune response, leading to hepatocyte necrosis, cell membrane rupture, and inflammatory responses. Additionally, inflammasomes play a crucial role in liver injury by promoting cytokine secretion, which attracts and activates macrophages and NEU. It is increasingly clear that the immune response during DILI is decisive, as T cell and immune system activation have been observed in DILI patients.^[18]^

Additionally, the immune response includes allergic reactions involving both the innate and adaptive immune systems’ inflammatory responses. A common mechanism is the activation of inflammasomes, where DAMPs activate inflammasomes in THP-1 cells. TNF-α enhances inflammation and directly damages hepatocytes, and TNF-αgene polymorphism −308G/A is significantly associated with drug-induced hepatitis (OR: 1.95, 95% CI: 1.11–3.14). DAMPs bind to toll-like receptors (TLRs), enhancing the immune response. For instance, a recent study showed that drugs forming new antigens with endogenous proteins can trigger adaptive immune responses, and certain human leukocyte antigen (HLA) polymorphisms increase the susceptibility of T cells to liver cell reactions.^[19]^ Similarly, in this study, CD4/CD8 levels were elevated in the drug-induced liver injury group, consistent with previous research findings.

Studies have shown that DILI caused by hypersensitivity reactions is associated with increased eosinophils and is characterized by elevated eosinophil levels in clinical presentations. Causality assessment for DILI (CDS) is a causality assessment tool for DILI that allocates points based on extrahepatic manifestations such as EOS. Further reports indicated that peripheral blood eosinophil levels have predictive value for detecting liver injury caused by anti-tuberculosis drugs. Our univariate and multivariate analysis shows that EOS% levels are elevated in patients with DILI, which is consistent with other research.^[20]^

Serum marker enzymes namely, ALT、AST、GGT、ADA are widely used to assess liver damage.^[21]^ While AST and ALT levels indicate hepatocellular injury, the severity is assessed by monitoring serum bilirubin and serum alkaline phosphatase^20^. These enzymes are released into circulation through necrosis of liver cells or membrane damage. ALT is more liver-specific and is, therefore, a better parameter for the diagnosis of liver damage. Comparison of baseline hematological parameters between the case group and the control group revealed that ALT, AST, GGT, and ADA have predictive significance for DILI.^[22]^

The current lack of specific biomarkers often leads to misdiagnosis and inappropriate treatment of DILI in these patients. Therefore, here is a critical need for DILI biomarkers that would improve early identification of DILI during drug-development, monitoring of DILI during clinical trials, early diagnosis in clinical practice and stratification of those who progress on to develop acute liver failure or develop chronicity in the longer term.^[20]^ Wang and others found that SII can reflect stronger inflammation and weaker immune response.^[23]^

Interestingly, we also found that NLR, MLR, SII, EOS%, and CD4/CD8 have predictive value for DILI in HBsAg-positive tuberculosis patients (*P* < .05). This indicated that SII was an independent risk factor for the occurrence of DILI in HBV-TB patients when the SII was <0.25. To our knowledge, this is the first study to investigate the correlation between SII and HBV-TB-DILI. In previous liver studies, the SII has often been used as a predictor of survival prognosis in patients with hepatocellular carcinoma or intrahepatic cholangiocarcinoma (ICC). Ren et al reported that SII could predict survival in ICC patients who received liver transplantation. Similarly, another study from China showed that SII is an effective prognostic factor for predicting outcomes in ICC patients undergoing radical hepatectomy.^[24]^ Furthermore, the ROC analysis in this study found that the combined prediction of HBV- TB patients developing DILI using SII along with NLR, MLR, EOS%, and CD4/CD8 yielded an AUC of 0.817. This predictive performance significantly outperformed that of any single indicator alone or any other combination in terms of diagnostic value. The combined application of these indicators enhances the predictive accuracy of the model for DILI occurrence in HBV- TB patients. The findings of this study align with the aforementioned conclusions, and the results show that SII demonstrates good performance in predicting the occurrence of DILI in TB patients with positive HBsAg. This is consistent with existing literature support. The SII and SIRI are recently defined inflammatory biomarkers associated with prognosis and treatment response.^[25]^

NLR、MLR、PLR were used as inflammatory markers in various studies for many diseases and drugs.^[26]^ The reason for this result is related to the functions of PLT, NEU, and LYM, and SII is related to inflammatory stress response and immune response intensity.^[15]^ Similarly, ALT, AST, ALP, and ALB have been reported as the hallmarks for determination, characterization, and severity grading for patients with DILI.^[27]^ Hepatocellular injury is characterized by an elevation of serum transaminases related to hepatocyte damage triggered by the toxin, and it is more likely to be associated with a poor outcome.^[18]^ However, our data indicate that there is statistically significant correlation between ALT and AST among different grades of DILI (*F* = 4.93, *F* = 6.079, *P* < .05, Table 3), which are associated with the prognosis of DILI. However, ALP, ALB, NLR, MLR, SII, EOS%, and CD4/CD8 show no statistical significance. Lack of any correlation between all inflammation-based parameters and the grading of DILI may be due to relatively good prognosis of our patients or sample size, which represents a major limitation of this single-center retrospective study with a relatively small sample size. This implies that the application value of inflammatory markers in assessing the severity of DILI may not be paramount.

Currently, there is no evidence suggesting that females have a higher overall risk for DILI, so sex is not a general risk factor for DILI.^[28]^ Data from the Spanish DILI Registry show a balanced sex distribution among DILI patients.^[29]^ However, some studies indicate that females may have a higher risk in specific situations, such as acute liver failure. Our study confirmed this, indicating a statistically significant difference in gender between the 2 groups, consistent with existing research showing an association between female gender and liver injury.

To sum up, our study aims to clarify that SII combined with NLR, MLR, EOS%, and CD4/CD8 has a good prognostic value for the occurrence of DILI in HBV- TB patients, which can provide guidance for clinical intervention. SII、NLR and MLR can be obtained directly through experimental testing, with low price, simple calculation, convenient operation, and no subjective factors involved. Therefore, our study may have better clinical practice implications for the diagnosis of TB-DILI.^[15]^

Although this investigation had a pragmatic approach, some limitations must be considered. Firstly, these results may not be directly applicable to other situation. Because this was a retrospective study, the liver function at the very beginning of anti-tuberculous treatment and other factors, such as the occurrence of single nuclear polymorphisms (SNPs) in the CYP2E1 gene or other drug-metabolizing enzymes could not be evaluated, as has been done in other articles.^[10]^ The absence of data exploring other factors previously associated with hepatotoxicity may result in confounding. Secondly, the number of cases in our study is limited, and the small sample size may affect the study results. This also makes it more difficult to extend our observations to a larger population. Finally, testing of prediction rules in different clinical scenarios is necessary to verify that results are valid and generally applicable.

## Acknowledgements

This study was supported by Science and Technology Program of Guangzhou (202201010744, 2024A03J0587, 2023A03J0539) and Guangzhou Medical Key Discipline (2021-2023,Tuberculosis).

## Author contributions

**Conceptualization:** Haiping Dong, Feng Liang, Yaping Dong.

**Data curation:** Haiping Dong, Jingyuan Feng, Xinwei Chang, Guidan Tang.

**Formal analysis:** Jingyuan Feng, Xinwei Chang, Weiming Fang.

**Funding acquisition:** Jingyuan Feng, Xinwei Chang.

**Investigation:** Jingyuan Feng.

**Methodology:** Jingyuan Feng, Guidan Tang, Haojie Tang, Weiyong Wang.

**Project administration:** Jingyuan Feng.

**Resources:** Jingyuan Feng, Weiming Fang.

**Software:** Jingyuan Feng, Weiming Fang.

**Supervision:** Shaoling Wu, Jinxing Hu.

**Validation:** Jinxing Hu.

**Visualization:** Feng Liang, Jinxing Hu.

**Writing – original draft:** Haiping Dong, Jingyuan Feng.

**Writing – review & editing:** Haiping Dong, Jingyuan Feng.
